# A remote sensing approach for exploring the dynamics of jellyfish, relative to the water current

**DOI:** 10.1038/s41598-023-41655-8

**Published:** 2023-09-07

**Authors:** Roee Diamant, Talmon Alexandri, Noga Barak, Tamar Lotan

**Affiliations:** 1 Department of Marine Technology, University of Haifa, Haifa, 3498838 Israel; 2 Department of Marine Biology, Leon H. Charney School of Marine Sciences, University of Haifa, Haifa, Israel

**Keywords:** Marine biology, Animal behaviour

## Abstract

Drifting in large numbers, jellyfish often interfere in the operation of nearshore electrical plants, cause disturbances to marine recreational activity, encroach upon local fish populations, and impact food webs. Understanding the dynamic mechanisms behind jellyfish behavior is of importance in order to create migration models. In this work, we focus on the small-scale dynamics of jellyfish and offer a novel method to accurately track the trajectory of individual jellyfish with respect to the water current. The existing approaches for similar tasks usually involve a surface float tied to the jellyfish for location reference. This operation may induce drag on the jellyfish, thereby affecting its motion. Instead, we propose to attach an acoustic tag to the jellyfish’s bell and then track its geographical location using acoustic beacons, which detect the tag’s emissions, decode its ID and depth, and calculate the tag’s position via time-difference-of-arrival acoustic localization. To observe the jellyfish’s motion relative to the water current, we use a submerged floater that is deployed together with the released tagged jellyfish. Being Lagrangian on the horizontal plane while maintaining an on-demand depth, the floater drifts with the water current; thus, its trajectory serves as a reference for the current’s velocity field. Using an acoustic modem and a hydrophone mounted to the floater, the operator from the deploying boat remotely changes the depth of the floater on-the-fly, to align it with that of the tagged jellyfish (as reported by the jellyfish’s acoustic tag), thereby serving as a reference for the jellyfish’s 3D motion with respect to the water current. We performed a proof-of-concept to demonstrate our approach over three jellyfish caught and tagged in Haifa Bay, and three corresponding floaters. The results present different dynamics for the three jellyfish, and show how they can move with, and even against, the water current.

## Introduction

Seasonal jellyfish blooms have a large impact on marine ecosystems^1^. The large aggregation of jellyfish affects fisheries, desalination and power plants, as well as public health and tourism^2,3^. Examining the swimming behavior of jellyfish could yield valuable insights into the mechanisms responsible for these blooms. Previous studies have shown that jellyfish possess a surprising range of swimming capabilities, including the ability to swim either with or against the current^4–7^. However, a significant obstacle that persists in measuring jellyfish swimming behavior lies in accurately assessing their motion relative to the water velocity in natural environments^8^. This work examines the motion of *Rhopilema nomadica*, which is the most common type of jellyfish in the East Mediterranean.

*Rhopilema nomadica* was first detected in 1976^9^. Within ten years, its populations increased tremendously and large annual swarms are now observed annually along the coast^10,11^. Large numbers of *Rhopilema nomadica* jellyfish penetrate the shore area in the summer (mostly July-Aug) and in winter (mostly Jan-Feb) months^12^, causing disturbances to marine infrastructures, and crowd out indigenous fish species, thereby interfering with the nearshore ecological balance. It is therefore of interest to explore *Rhopilema nomadica’s* migration patterns and, specifically, to better understand its motion mechanism with respect to the water current.

In this work, we propose a tagging-based method to track the short-term trajectory of jellyfish, relative to the water current’s velocity field with zero-to-low disturbance to the animal’s motion. Tagging jellyfish is a useful tool for studying their behavior, swimming capabilities, migration patterns, and population dynamics. Different technologies have been applied for jellyfish tagging (for a thorough review, see^13^). The most common methods are based either on attaching a cable tied loosely between the jellyfish’s bell and its oral arms^6^, or by gluing the tag to the jellyfish’s bell^14^. Having the tag attached on the pulsating bell holds the advance of reducing the distortions of the signal from the tag to the receiver. In particular, due to the non-negligible acoustic target strength of the jellyfish^15^, acoustic signals passing through its body encounter multipath and phase distortions. In terms of acoustic reception, instead of within or underneath it, it is therefore better to place the tag on the bell, where the tag’s emitted signals encounter less distortions. The tag may be an acoustic transmitter and may contain a time-depth recorder and various sensors, such as for light, temperature, acceleration, and pressure^7,16,17^. In order to track the jellyfish’s motion, the tag is attached via a cable to a surface floater for position reference and for retrieval^13^. For longer-term tracking in deep water, tags serve as data loggers and are programmed to ascend to the surface and transmit the data through radio or satellite^6,17,18^. However, to the best of our knowledge, no solution has been proposed thus far that accurately measures jellyfish dynamics with respect to the water current. In particular, having a surface buoy attached to the jellyfish may induce drag and influence the jellyfish’s motion. Furthermore, the water current experienced by the jellyfish at depth is not measured.

To evaluate the jellyfish’s motion relative to the water current with minimum disturbance, we take a remote sensing approach and simultaneously measure the jellyfish’s trajectory estimation and the water current velocity field. For the former, we rely on the acoustic tag attached to the jellyfish’s body. These are small (70 mm long) devices, which periodically emit acoustic signals that encode the tag’s ID number and its current depth. Jellyfish come in many shapes, sizes, and colors but share an umbrella-like body, known as a *bell*. The bell is fully exposed to the sea water and remains relatively stable as the jellyfish moves. Hence, we attach the acoustic tag to the bell’s upper part using a special type of glue. Being large in size, with a body diameter that may reach up to 40 cm, the *Rhopilema nomadica* is ideal for tagging by gluing the tag to the jellyfish’s bell. In addition, since this jellyfish size is roughly an order of magnitude larger than the transmitter, hydrodynamic disturbance by the transmitter that may affect the jellyfish swimming are reduced.

Acoustic tagging is a widely used technique for tracking marine fauna^19^. Acoustic tags have been used to examine the behavioral and social interactions of animals like sharks, sea turtles, and seals^20,21^. The signals from the acoustic tags are detected and decoded by several remote acoustic recorders that measure the time-of-arrival (ToA) of the tag’s emissions and decode the tag’s ID and depth^22^. The measured ToAs of the tags’ signals are then used for the localization of both the jellyfish and the floater. After time synchronizing the recorders, the measured ToA are grouped into pairs, while taking advantage of the fixed time interval (TI) and multiple reception by different recorders to yield time-difference-of-arrival (TDoA) samples. Furthermore, merging TDoA measurements yields a hyperbola for the locations where the animal may be. The intersection of at least two hyperbolas provides a unique 2D localization solution^23^. The case of too few receptions is handled by using the target’s motion^24^, by fitting a motion model^25^ or by tracking^26^.

To evaluate the water current, we constructed an autonomous Lagrangian floater that freely drifts with the water current, while maintaining a pre-defined or an on-demand depth by turning on or off a thruster^27^. The floater is deployed together with the tagged jellyfish, and its trajectory is then evaluated by attaching another acoustic tag below its thruster. The evaluated trajectories of the jellyfish and the floater are compared to draw conclusions about the jellyfish’s motion with respect to the water current, as represented by the floater’s time-varying location. In an attempt to serve as a reference for the water current experienced by the jellyfish, the floater’s depth is set on-demand after its deployment by changing its target depth on-the-fly. This is made possible via an on-boat, underwater modem and on-floater hydrophone pair. Specifically, a processor on the boat decoded the jellyfish’s depth by the emissions of its tag, modulated the depth information into a communication message, and acoustically transmitted this information to the floater to remotely synchronize the depth of the floater with that of the jellyfish. In this process, the maximum time delay was 20 s from the time of tag’s emission until the floater received the message. Then, the change in the floater’s depth occurred after a maximum of roughly 25 s for a total leg time of 45 s. For stability, we allowed this change in the floater’s depth to occur only once. We note that in this process we assume the floater and jellyfish experience the same water current. Our method is thus sensitive to local turbulence, especially when, in time, the floater and the jellyfish may spread apart.

## Results

We provide a proof-of-concept to demonstrate our method for comparing the motion of jellyfish to the water current in three tagging operations. These operations involved three jellyfish and three corresponding floaters. The results are shared below.

### Description of the testbed

The area chosen for the deployment is Haifa Bay (32.846428, 34.998581), where the *Rhopilema nomadica* jellyfish are abundant. The area is characterized by significant water currents with local turbulence, making it interesting for the short-term tracking of jellyfish motion, relative to the water current. The area is relatively flat, and muddy-bottomed with a water depth ranging from 20 to 25 m.

**Figure 1:**
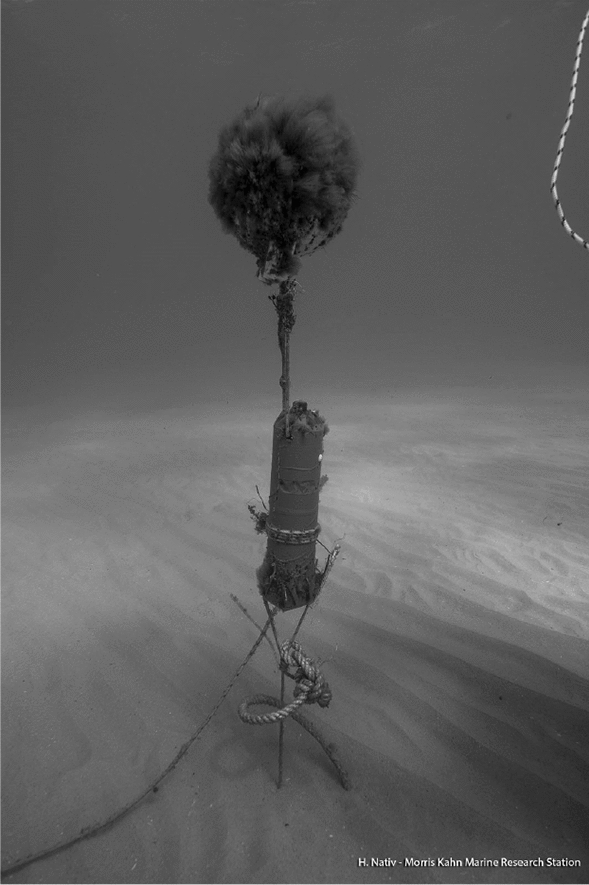
Picture of one of the deployed receivers.

**Figure 2:**
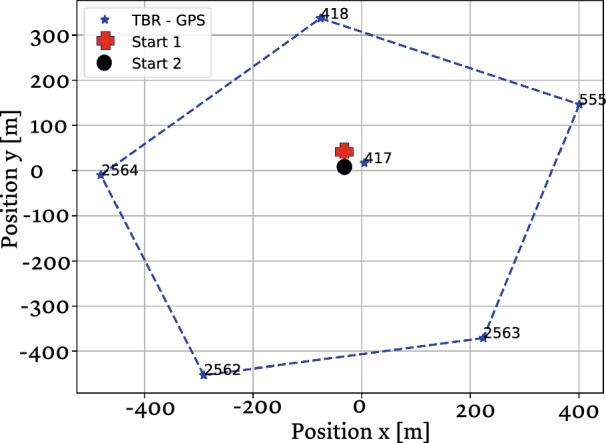
Deployment area of the July 2022 experiment with starting points 1 and 2 marked close to the central receiver. Numbers at the vertices represent different receivers.

Our testbed included the deployment of six acoustic receivers in an area of 0.45 ${\text{km}}^2$. Using a submerged float, each of the receivers was stationed pointing up roughly 1 m above the seabed, as shown in Fig. 1. Each recorder included a temperature sensor that allowed for the calculation of the sound speed in water^28^. Based on the seabed bathymetry and the measured sound speed profile, we used the method in^29^ to determine the optimal position of the receivers with a single receiver in the middle, such that the receivers are separated by roughly 500 m (see Fig. 2). Releasing the jellyfish and floater in the middle of this structure, we guarantee reception by at least four receivers regardless of the water current’s direction. In Fig. 2, the indication for start of measurement refers for both the jellyfish and the floater as both where released at the same time.

#### Description of the acoustic tags and tagging operation

We used the DR-HP16 model acoustic tags from Thelma Biotel Inc. These are 70 mm-long tags, which weigh 14.9 g in water. The tags generate short emissions in the carrier frequency of 69 kHz of roughly 100 msec long at a source power level of 158 dB//$1\,\upmu \hbox {Pa}$@1m, and their detection range was measured at roughly 1000 m in a test range^30^. The tags were programmed to transmit their ID number and depth in a fixed TI, ranging from 30 to 45 s, thereby reducing the chances of signal loss due to collisions at the receivers. A byte that identifies the sequential number of the data packet is transmitted to each tag’s emission. This mechanism allows for the identification of the tag reception and corresponding ToA for the same tag emission at the different receivers. The low source exposure level of the tags is well below the limitations set for sound exposure in water^31^. Further, the tags are programmed to stop emitting automatically after a signal day (programmable), which further reduces the exposure level.

To attach the acoustic tag to the jellyfish’s bell, we used Histoacryl glue, a topical skin adhesive glue which is also used for tissue recovery. This type of glue has proven effective for non-rigid surfaces directly exposed to salt water^32^. The procedure involved applying the glue over the tag’s surface and holding the tag against the jellyfish bell for at least 30 s. Experiments performed in the lab showed that, this way, the tag remains attached to the jellyfish for at least three hours.

#### Details of the submerged floater

The submerged floater made for the jellyfish tracking operation was a self-made sealed perspex tube to which a thruster is attached, along with a depth sensor. The floater is made to be roughly Lagrangian with very low drag in the horizontal plane (a more detailed description is provided in the “Methods” section). On its vertical plane, the thruster is controlled by an Arduino controller which turns the thruster on and off to control the floater’s depth. This operation allows depth profiling between 1 and 100 m, and depth keeping at a range of 0.5 m. A hydrophone attached to the floater continuously listens for possible commands transmitted from the deploying boat to change the floater’s depth on-the-fly. Relying on depth information acquired by decoding the transmissions from the jellyfish’s tag, we are able to match the floater and jellyfish depth to obtain a more reliable reference for the water current experienced by the jellyfish. Using a set of batteries, the floater is made to operate for a few hours, during which its position is tracked by another acoustic tag tied below its structure.

#### The collected dataset

**Figure 3:**
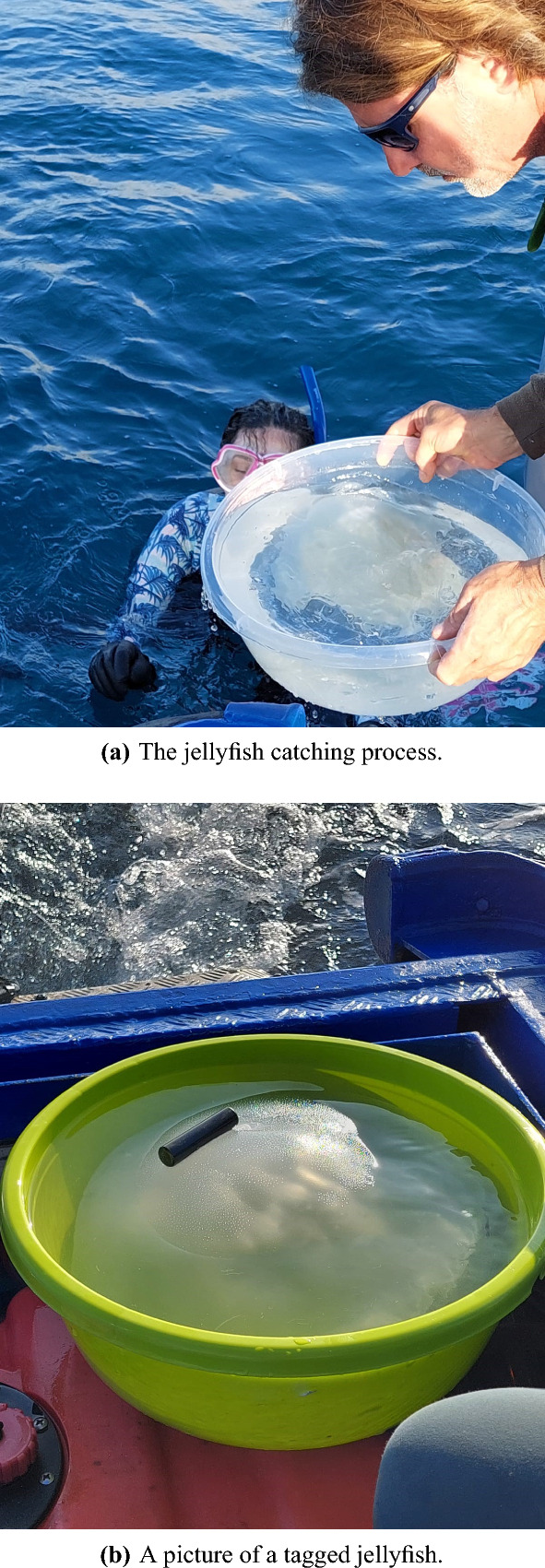
Pictures from the July 2022 tagging activity. The authors give their full consent for publication of identifying information/images in the online open-access publication.

Jellyfish were collected manually. After deploying the six recorders, a boat surveyed the area to find jellyfish. This procedure was performed in the early morning when the jellyfish are expected to be close to the surface. Once a jellyfish was identified, a swimmer caught it using a 12-liter round bucket to avoid damage to the animals. The bucket with the jellyfish and sea water was then moved to the vessel, where the jellyfish was tagged. After tagging, the boat moved to the center of the receiver deployment area to achieve better acoustic coverage when releasing the jellyfish. The jellyfish’s release was performed at the same time as that of the reference floater. A swimmer then followed the released jellyfish for a few minutes to verify that the acoustic tag had remained glued to the jellyfish’s bell, and to verify that the jellyfish kept swimming. In total, three *Rhopilema nomadica* jellyfish were tagged in two deployment actions performed in February, 2022 and July, 2022. Figure 3 shows pictures from one of these deployments, demonstrating how the jellyfish was caught and how the tag was glued to the jellyfish’s bell.

From each deployment, we collected a set of ToA measurements measured by each of the six deployed receivers.

The ToA measurements were time-synchronized relative to those of the center receiver. This was preformed by attaching a *sync* tag to the center receiver. The sync tag emitted message with an incremental value once in roughly 10 min. The corresponding ToA at receiver *i* can be written as

$$\begin{aligned} \text {ToA}_i=\frac{r_i}{c}+\Theta _i, \end{aligned}$$

where $r_i$ is the distance between Receiver *i* and the center receiver, *c* is the sound speed in water, and $\Theta _i$ is the bias of receiver *i*’s clock relative to that of the center receiver. The latter is because the tag is attached to this receiver and so its ToA is 0. Since $\text {ToA}_i$ is measured, *c* is calculated by temperature readings at the receivers, and $r_i$ is known, $\Theta _i$ can be estimated periodically for every emission of the sync tag. See further details in^22^. After time synchronizing the ToA measurements at each receiver, we divide the set per tag ID and combine readings from pairs of receivers to yield TDoA measurements. Intersections between the TDoA are merged in a least square framework to obtain position estimates. Finally, expecting smooth trajectories for both the jellyfish and floater, we remove outliers whose regression error is significantly higher ($|r|>0.7$) than a polynomial fit of degree 3 for the resulting positions. The measured positions enable the estimation of the heading and the speed for both the jellyfish and the floater.

### Localization results

**Figure 4:**
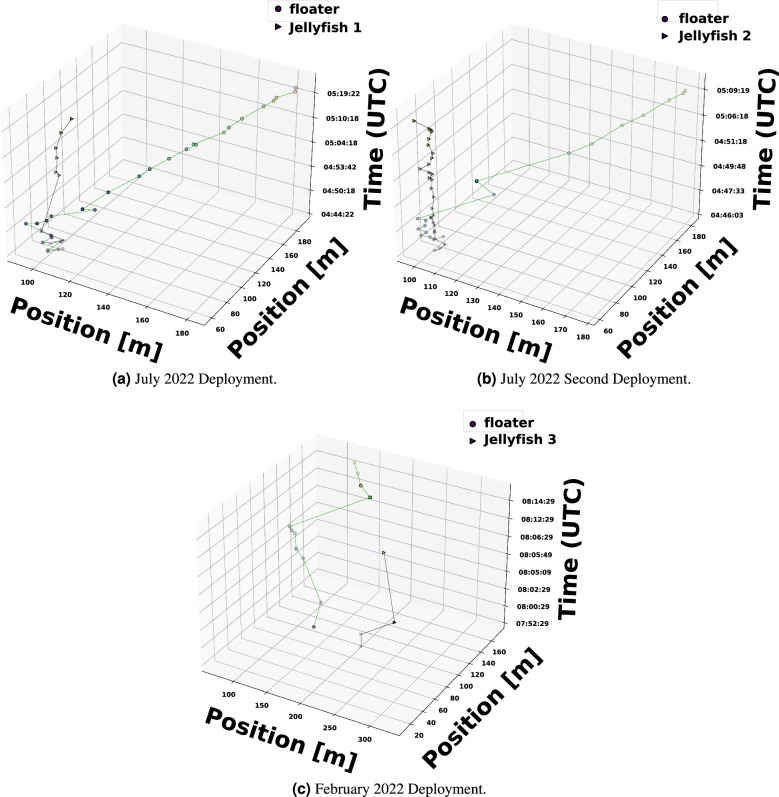
Zoom-in on the two July 2022 deployments and on the February 2022 experiment. Experiment time appears as the z axis.

In Fig. 4, we show the trajectories of the three floaters and jellyfish in the three deployment exercises. We note that outliers were filtered out. These outliers were identified as positions well beyond the expected smooth trajectory of the float and jellyfish. We observe that in all three experiments, both the floater and the jellyfish moved in different patterns. For example, in the July experiment the floaters progressed with the water current, while the jellyfish remained close to the starting point. In contrast, in the February experiment the jellyfish progressed in the opposite direction of the drifter. This latter motion characteristic was consistent throughout the detection period of over one hour, which reflects the jellyfish’s ability to overcome the water current in regard to its motion. From Fig. 4, we observe that, during the measurement time frame, the floater and jellyfish drifted apart. Naturally, the correlation between the water current they experience reduces with that drifting distance. While this correlation cannot be estimated without a model for the water current, it can be bounded using the Rossby radius of deformation that is effected by the earth’s Coriolis force parameter and the local latitude^33^, and which bounds the coherence distance for the water current.

**Figure 5:**
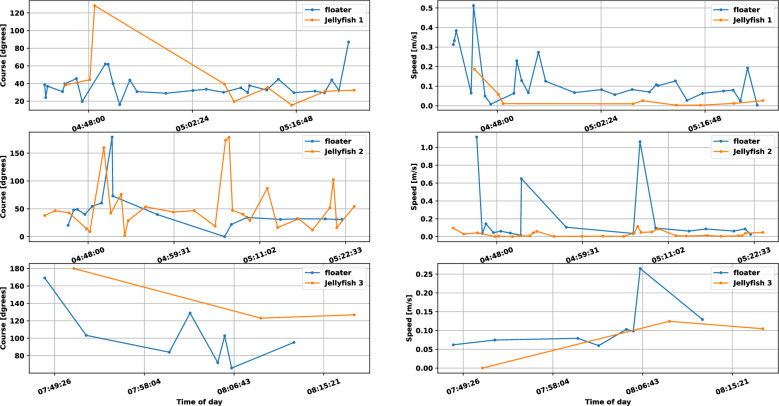
The instantaneous speed and course of the floaters and jellyfish during the three deployments. Time is shown in UTC, and the local time during experiments was UTC+2.

**Figure 6:**
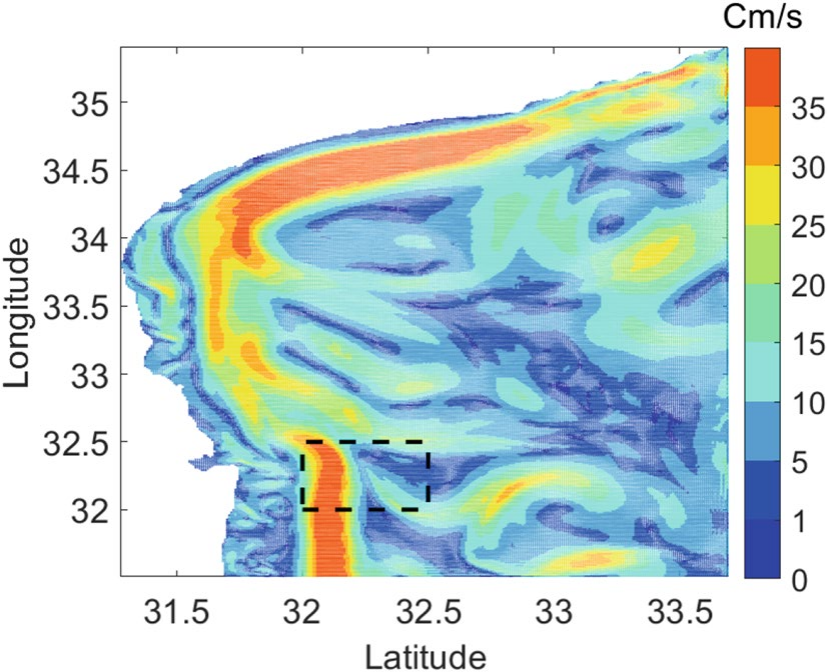
Map of velocities for the area explored in the jellyfish measurements. Deployment area is marked by the black rectangular. Example produced from the SELIPS model^35^. Values in the map are seasonally averaged flow velocities.

Next, we explore the velocity differences among the three jellyfish-floater pairs. In Fig. 5, we show the instantaneous speed and heading direction (course) of the jellyfish and their corresponding floaters as calculated by the estimated locations for both. Time is shown in UTC, and the local time was UTC+2. We observe that the speed and course of Jellyfish #2 roughly matched that of their accompanying floater, with several mutual transients in the course measurement that may have resulted from local turbulence. However, the heading direction of Jellfyfish #1 (at the beginning of the measurement) and #3 did not match their corresponding floaters. The speed of Jellfyfish #3 and partially of Jellfyfish #2 matched their accompanied floater, but a significant difference is shown for Jellfyfish #1. Figure 5 shows outliers that reach 1 m/s. One explanation for these high values are non-line-of-sight multipath mistakenly regarded as line-of-sight. As discussed in^34^, this confusion can lead to significant localization error. When such error precedes an accurate localization estimation, seemingly high velocities are calculated and appear as outliers. This explanation is supported by the fact that much less outliers are visible for the jellyfish track. In particular, since the jellyfish’s tag is attached to the bell, less seabed-multipath arrive to the receiver compared to that of the floater whose tag is completely exposed to the sea water. As a result, the expected number of cases where non-line-of-sight are mistaken for the line-of-sight is lower for the jellyfish. The instantaneous changes in the speed and course are explained by the existence of turbulence in the area explored. To support this claim, we include in Fig. 6 the outcome of the SELIPS ocean current model^35^ for the explored area predicting non-negligible turbulence in the water current. The model shows values in agreement with the velocities obtained, as well as turbulence.

Results of a linear regression over the above results are given in Table 1 for both the speed and course. Here we also observe that, on average, Jellyfish #2 roughly maintained the speed and heading direction of the water current, as reflected by its speed and course similarities to that of its accompanying floater. However, the significant differences between the velocities of Jellyfish #1 and #3, relative to their corresponding floaters, shows that these jellyfish did not explicitly follow the water current. This is also supported by the differences in the root mean square error (RMSE) between the instantaneous course and the linear regression one of the jellyfish and floater as shown in Table 1. A reason for the observed differences between the median and regression speeds are small changes in the coarse of the jellyfish, which do not affect the trajectory but do impact the median value.

**Table 1:** Momentary median speed and linear regression (LR) results for the speed and course of the three jellyfish-floater pairs.

Date	Subject	Median speed (m/s)	LR speed (m/s)	Course (°)
July 22 Exp. 1	Jellyfish 1	0.054	0.003	85.8
	Floater	0.107	0.049	39.6
Jul 22 Exp. 2	Jellyfish 2	0.011	0.03	43.2
	Floater	0.091	0.052	37.6
February 22	Jellyfish 3	0.104	0.044	122.6
	Floater	0.129	0.07	64.2

## Discussion

The performed experiments for proof-of-concept included only three jellyfish, which may be a too-small dataset to reliably conclude about jellyfish motion. However, the results demonstrate how the proposed tracking approach can track the motion of jellyfish relative to the water current in 3D. We note that, besides understanding the motion of jellyfish with respect to the water current, the concept of relative tracking of single jellyfish can also be exploited to explore other questions. For example, in a future work we plan to use our approach to tag and track jellyfish within a swarm, while tracking the swarm advance with drones and satellites, thereby better understanding how individual jellyfish move within the swarm. Another future direction to overcome the distance limitation of the fixed receivers for long-term jellyfish tracking is to use a surface vehicle following the emissions from the jellyfish’s tag from above, while carrying an acoustic Doppler current profiler (ADCP) for water current measurements. A possible tool for such surface following is the autonomous surface vehicle (USV) described in^36^, which operates using solar power and can activate acoustic detection processes.

Our method for tracking both jellyfish and floater has the advantage of minimal disturbance to the animal, while obtaining reliable reference regarding the water current’s velocity field. Using the acoustic modem-hydrophone pair, we could also change the depth of the floater on-the-fly to fit that of the jellyfish, thus ensuring a similar environment for both. The method is geared towards short-term analysis and enables 3D tracking. The main benefit of our approach is its remote sensing solution, where the tracking is performed by a distant set of receivers and there is no need to recover the tagged jellyfish for data collection. Comparing the estimated trajectory of the jellyfish with that of the floater, the novelty of our approach is its ability to explore the small-scale dynamics of the jellyfish, while accounting for the effect of the water current on the jellyfish’s motion.

One disadvantage of our method lies in the cost of the acoustic tags, which is hard to recover after the operation. This limits the number of tagged jellyfish, and, as a result, makes it difficult to obtain sufficient information for tasks such as following a jellyfish swarm. A possible way to solve this problem would be to deploy a floater among the swarm, while actively detecting the neighboring jellyfish. In addition, previous works have shown that jellyfish can be detected acoustically by transmitting wideband signals and analyzing the reflections received^37^. By tracking such reflections while drifting with the water current, the floater can evaluate the relative motion of several jellyfish simultaneously, without the need for tagging. The task, however, is challenging, as the floater needs to carry multiple hydrophones and remain stable in the water column to employ, e.g., beamforming for source separation.

To conclude, our proposed approach is a remote sensing solution to enable the tracking of jellyfish motion with respect to the water current with very little to none interference to the animal. The tracking is performed for a short period of time to examine small scale motion changes in 3D. Our proof-of-concept results present different dynamics for three jellyfish, and show how they can move with, and even against, the water current. The approach can be further used to track the motion of individual marine animals within a swarm.

## Methods

### Floater structure

**Figure 7:**
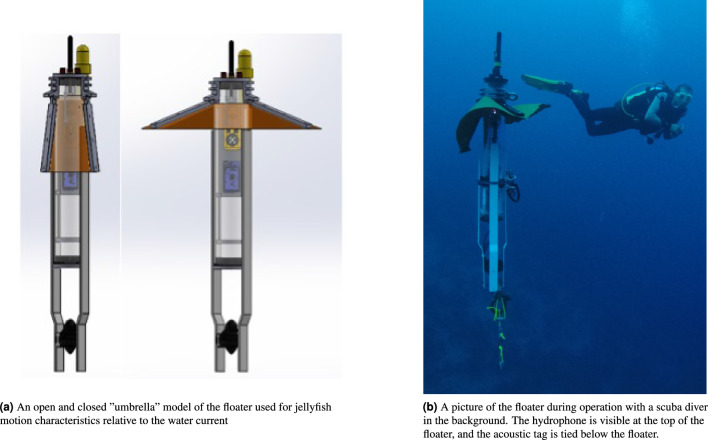
The Lagrangian floater: model and picture.

The floater used for the jellyfish motion characterization task is a 3-inch cylinder to which we attach a hydrophone for decoding changing depth commands; a pressure sensor to monitor the floater’s depth; salinity and temperature sensors for evaluating the sound speed in water; a thruster for the floater’s ascension; weights to balance the floater to be 100 g negative in water; and an acoustic tag. See illustration in Fig. 7a. Controlling the thruster is an Arduino card that switches the thruster on and off, based on the floater’s depth. In particular, to maintain the floater around a target depth *d*, the thruster is activated once a lower limit, $d+\Delta ^{\text{l}}$, is reached, and deactivated once an upper limit is reached, $d-\Delta ^{\text{u}}$. In our trials, we managed to keep the floater traversing 0.5 m around the designated depth. Assisting in this operation is a flexible fabric strapped to the floater’s tube that, like an umbrella, opens upon descending using the water’s drag force, and closes upon ascending. This fabric is used to reduce the time the thruster is activated by more than half, thereby reducing the power consumption. The recovery of the floater is performed by programming it to surface after a designated time. A safety weight-drop mechanism, based on a dissolved metal ring, ensures surfacing in the case of failure. The floater is made to be roughly Lagrangian, i.e., to float within the water current with minimal drag. A series of in-situ experiments proved this characteristic^27^. A picture of the floater during operation is shown in Fig. 7b.

The hydrophone attached to the floater is sampled by a TLV320ADC6140 sound card connected to a Raspberry-Pi 3 controller. The controller continuously runs a decoder for possible messages transmitted acoustically by the deploying vessel. These messages are interpreted by the floater as change-of-mission for e.g., depth change or surfacing. In the context of the jellyfish tracking, this underwater acoustic communication is used to match the floater’s depth to that of the jellyfish, thereby ensuring that both floater and jellyfish experience the same water current. In particular, based on the jellyfish tag’s signalling, the operator on the vessel can acoustically direct the floater to meet the same depth. The underwater acoustic communication is based on the Janus standard^38^ for short message transmission.

### Acoustic localization of the jellyfish and floater

#### Reception by at least 3 receivers

The acoustic localization of the tags is performed based on the ToA measurements detected by a subset of the $R=6$ receivers. These are merged into TDoA measurements by the intersection of at least two iso-lines in a 2D plan. For its emission at time instance *t*, denote the 2D UTM position of tag *i* as $\textbf{s}^i_{{\text{tag}}}(t)=[x^i_{{\text{tag}}}(t), y^i_{{\text{tag}}}(t)]$, and the 2D UTM position of receiver *n* as $\textbf{s}^{\text{n}}_{{\text{rec}}}=[x^n_{{\text{rec}}}, y^n_{{\text{rec}}}]$. Note that the receivers are anchored and therefore $\textbf{s}^{\text{n}}_{{\text{rec}}}$ is fixed. The distance between receiver *n* and the *i*th tag is defined as $\textbf{d}_{i,n}(t)=|\textbf{s}^n_{{\text{rec}}}-\textbf{s}^i_{{\text{tag}}}(t)|$. Neglecting the tag’s sub-index and time instance *t* for simplicity, the iso-line corresponding to the TDOA between receivers *n* and *k* is $\mathbf{\rho }_{\text{n,k}}=\textbf{d}_{\text{n}}-\textbf{d}_{\text{k}}$. The relation of the latter and the time synchronized TDoA measurement, $\tau _{n,k}$, is

1 $$\begin{aligned} |\mathbf{\rho }_{n,k}|+e_{\text{n,k}}=\tau _{n,k}\cdot c+e_{\text{n,k}}, \end{aligned}$$

where *c* is the sound speed in water and $e_{n,k}$ is a measurement noise of $\tau _{n,k}$.

For all pairs of receivers *n*, *k* detecting the same emission of tag *i*, we minimize a utility function,

2 $$\begin{aligned} \hat{\textbf{s}}^i_{{\text{tag}}}=\underset{\textbf{s}^i_{{\text{tag}}}}{{\text{argmin}}} \sum _n\sum _k|\textbf{d}_{\text{n,k}}-\textbf{s}^{\text{i}}_{{\text{tag}}}|. \end{aligned}$$

Finding the intersection of the iso-lines is a nonlinear optimization problem^39^. Furthermore, due to uncertainties in receivers’ positions and due to measurement noise, the intersection of iso-lines is not likely to converge to a single point^40^. Thus, it is important to utilize additional available information when more than three receivers are detecting the same tag’s emission. In particular, we use the ordinary least squares (OLS) approach.

We start by defining one of the receivers as a reference receiver, whose 2D UTM position is set at $\textbf{s}^0_{{\text{rec}}}=[x^{0}_{{\text{rec}}}, y^{0}_{{\text{rec}}}]$. Given the TDOA between the $n$th receiver and the reference receiver, $\tau _{\text{0,n}}$, we obtain

3 $$\begin{aligned} \tau _{\text{0,n}} = \frac{1}{c}\left( |\textbf{s}^{\text{i}}_{{\text{tag}}} - \textbf{s}_0| - |\textbf{s}^{\text{i}}_{{\text{tag}}} - \textbf{s}_{\text{n}}| \right) = \frac{1}{c}\left( \textbf{d}_{\text{0}} -\textbf{d}_{\text{n}}\right) . \end{aligned}$$

Let the distance difference of arrival for the *n*th receiver be

4 $$\begin{aligned} \textbf{D}_{\text{0,n}} = \tau _{\text{0,n}} \cdot c = \textbf{d}_{\text{0}} -\textbf{d}_{\text{n}}, \end{aligned}$$

such that

5 $$\begin{aligned} \textbf{d}_\text{0}^2 -\textbf{d}_\text{n}^2 = |\textbf{s} - \textbf{s}_0|^2 - |\textbf{s} - \textbf{s}_\text{n}|^2 = 2\textbf{d}_0 \textbf{D}_\text{0,n} - \textbf{D}_\text{0,n}^2. \end{aligned}$$

Denote

6 $$\begin{aligned} b_\text{n} = \frac{1}{2}\left( [x^0_{\text{rec}}]^2+[x^n_{\text{rec}}]^2+ [y^0_{\text{rec}}]^2+[y^n_{\text{rec}}]^2+\textbf{D}_\text{0,n}^2\right) , \end{aligned}$$

which, after rearranging, becomes

7 $$\begin{aligned} b_\text{n}=(x^0_{\text{rec}}-x^n_{\text{rec}})x + (y^0_{\text{rec}}-y^0_{\text{rec}})y + \textbf{D}_\text{0,n}{} \textbf{d}_0. \end{aligned}$$

This is a linear model for the unknown tag position *x*,  *y* and the range $\textbf{D}_0$. In a matrix form, for *N* receivers detecting the same tag’s emission, we obtain

8 $$\begin{aligned} \textbf{A}{} \textbf{X} = \textbf{b}, \end{aligned}$$

where

9 $$\begin{aligned} \textbf{A} = \left[ \begin{array}{ccc} x^0_{\text{rec}} - x^1_{\text{rec}} &{} y^0_{\text{rec}}-y^1_{\text{rec}} &{} \textbf{D}_{0,1} \\ x^0_{\text{rec}} - x^2_{\text{rec}} &{} y^0_{\text{rec}}-y^2_{\text{rec}} &{} \textbf{D}_{0,2} \\ &{} \vdots &{} \\ x^0_{\text{rec}} - x^N_{\text{rec}} &{} y^0_{\text{rec}}-x^N_{\text{rec}} &{} \textbf{D}_\text{0,N} \end{array} \right] , \end{aligned}$$

$\textbf{X}=\left[ x^i_{\text{tag}},y^i_{\text{rec}},\textbf{d}_0\right] ^\text{T}$, and $\textbf{b}=\left[ b_0,\ldots , b_n\right] ^\text{T}$. The OLS solution for (8) is obtained by

10 $$\begin{aligned} \textbf{X}_\text{ols} = \left( \textbf{A}^\text{T}{} \textbf{A}\right) ^{-1}{} \textbf{A}^\text{T}{} \textbf{b}. \end{aligned}$$

#### Reception by less than 3 receivers

In the under-ranked case, where the tag’s emissions are decoded by less than three receivers, the above procedure yields ambiguities. To solve these, we rely on the constant TI between each tag’s emissions as well as on the assumption that both the jellyfish and the floater move at a fixed (unknown) velocity, $v_x,v_y$.

For time instance *t*, the time-of-flight (ToF) is

11 $$\begin{aligned} \rho ^i_n(t)=\frac{1}{c}\sqrt{(x^i_{\text{tag}}(t)-x^n_{\text{rec}}(t))^2 +(y^i_{\text{tag}}(t)-y^n_{\text{rec}}(t))^2}. \end{aligned}$$

Denote the fixed interval between the emissions from each tag *i* as $\text{TI}^i$. For the same tag *i* and receiver *n*, consider the ToA of consecutive measurements collected at time *t* and $t+\text{TI}^i$, $\text{ToA}^i_n(t)$ and $\text{ToA}^i_n(t)+\text{TI}^i$, respectively. The TDoA is

12 $$\begin{aligned} \theta ^i_n(t)= & {} \text{ToA}^i_n(t)+\text{TI}^i-\text{ToA}^i_n(t)\nonumber \\= & {} \rho ^i_n(t+\text{TI}^i)-\rho ^i_n(t)+\text{ToA}^i. \end{aligned}$$

We consider the state space $S(t)=\{x^i_{\text{tag}}(t),y^i_{\text{tag}}(t),v_x,v_y\}^T$, and formalize the relation between *S*(*t*) and $S(t+\text{TI}^i)$ by

13 $$\begin{aligned} S(t+\text{TI}^i) = F\cdot S(t)+B(t)\cdot U(t+\text{TI}^i) + N, \end{aligned}$$

where *F* is a fixed transition matrix that depends on the TIs, *B*(*t*) is the measurement matrix that depends on $\theta ^i_n(t)$, *U*(*t*) is the data vector that encompasses the state variables, and *N* is a measurement noise matrix. The solution is found by minimizing the utility function

$$\begin{aligned} \sum _{m=1}^M\left( \hat{\theta }^i_n(t_m)-\theta ^i_n(t_m)\right) , \end{aligned}$$

for *M* measurements, where $t_m$ is the time instance for the $m$th measurement, and $\hat{\theta }^i_n(t_m)$ is a calculated TDoA based on the estimated position of the tracked tag. Further details are available in^41^ (Supplementary Information S1).

### Evaluating the trajectory’s parameters

Once the time-varying positions of the jellyfish and floater are calculated, we can evaluate their similarities by comparing the speed and heading of their trajectory. The momentary speed is obtained by differentiating consecutive estimated locations and dividing by the difference between the number of TI passed between the two emissions, where the latter is known by the accumulated packet number encoded on each tag’s emission. Similarly, the course is obtained through linear regression over the complete set of positions with respect to the north. To avoid outliers, the results are smoothed using, e.g., a median filter.

### Supplementary Information

Supplementary Information.

## Data Availability

The datasets generated and analysed during the current study are available in the jellyfish repository: https://drive.google.com/file/d/1TqBq4hhJ2az7fBkh7w5vNEiGVnLyQg11/view?usp=sharing. All data generated or analysed during this study are included in this published article [and its supplementary information files].
