# Supplementary material for: A remote sensing approach for exploring the dynamics of jellyfish, relative to the water current
Source: Sci Rep. 2023 Sep 7;13:14769. doi: 10.1038/s41598-023-41655-8 (PMC10485037; doi:10.1038/s41598-023-41655-8)
Supplement: Supplementary file 1 — Supplementary Information. [file 41598_2023_41655_MOESM1_ESM.zip › Coordinates of TBRs for 17 Feb 2022.docx]

**Coordinates of TBRs for 17/2/2022 experiment**

| # | TBR | Coordinates | | Remarks | Dror/Talmon |
| --- | --- | --- | --- | --- | --- |
| 1 | 1155 | N32^0^51.083' | E34^0^58.697' | Position at retrieval Includes sync tag | Dror |
|  |  | N32^0^51.2939' | E34^0^58.4662' |  | Talmon |
| 2 | 1153 | N32^0^51.092' | E34^0^58.145' | Waypoint #27 | Dror |
|  |  | N32^0^51.2857' | E34^0^57.9954' |  | Talmon |
| 3 | 417 | N32^0^51.073' | E34^0^58.128' |  | Dror |
|  |  | N32^0^51.1219' | E34^0^58.2202' |  | Talmon |
| 4 | 1154 | N32^0^50.725' | E34^0^58.211' |  | Dror |
|  |  | N32^0^50.9805' | E34^0^57.9576' |  | Talmon |
| 5 | 418 | N32^0^50.724' | E34^0^58.683' | First deployed TBR | Dror |
|  |  | N32^0^50.9432' | E34^0^58.3498' |  | Talmon |
