# Supplementary material for: A remote sensing approach for exploring the dynamics of jellyfish, relative to the water current
Source: Sci Rep. 2023 Sep 7;13:14769. doi: 10.1038/s41598-023-41655-8 (PMC10485037; doi:10.1038/s41598-023-41655-8)
Supplement: Supplementary file 1 — Supplementary Information. [file 41598_2023_41655_MOESM1_ESM.zip › Meta_220719_jellyfish.docx]

Jellyfish 19.07.2022 experiment eta data

(You may check the planned deployment for reference)

Times in this document are local time. Times in the csv files are UTC (but local time when displayed by comport of screen is defined as UTC + 2 and not UTC + 3, so no summertime in comport. The csv file is UTC though)

TBRs depths approx. 5 meter above seabed

| time | TBR # | Lat Lon – Talmon | Lat Lon Roee | Water Depth [m] |
| --- | --- | --- | --- | --- |
| 06:44 | 2563 | 32^0^51.4054' 034^0^58.7526' | 32.85703 34.97915 | 20 |
| 06:53 | 2562 | 32^0^51.4668' 034^0^58.4372' | 32.85775 34.97391 | 20.1 |
| 06:58 | 2564 | 32^0^51.7081' 034^0^58.3211' | 32.86207 34.97193 | 19.9 |
| 07:03 | 418 | 32^0^51.8917' 034^0^58.5847' | 32.86492 34.97641 | 19.9 |
| 07:10 | 555 | 32^0^51.7839' 034^0^58.8878' | 32.86311 34.98146 | 19.9 |
| 07:15 | 417 | 32^0^51.7177' 034^0^58.6323' | 32.86195 34.97721 | 23.2 |

Drifter 2 – tags 75, 81 depth profile: 4-5 m

Drifter 4 – tags 77, 79 depth profile: 10-12 m

Jellyfish 1 (big) – tag 89 deployed with drifter 2 at 07:41 at 32.86202 34.97681

Jellyfish 2 (small) – tag 83 deployed with drifter 4 at 07:42 at 32.86212 34.97680

Water temp 28.8^0^C - average of TBR sensors during exp. Time (in agreement with SVP taken during the experiment time separately)

TI = [70, 70, 80, 80, 90, 90, 0, 48, 56, 56, 80, 80, 600, 600, 600]

In an ascending order of tag numbers

Tx interval - 0 means random (or I couldn't figure it out properly)

Note the difference between TBR measured sound velocity based on TDoA measurement to the SVP taken on site (Wilson formula) 1521 vs. 1548. We assume this is due to inaccuracies in the GPS position and measurement noise of the TBRs.
